# Unknown Object Detection Using a One-Class Support Vector Machine for a Cloud–Robot System

**DOI:** 10.3390/s22041352

**Published:** 2022-02-10

**Authors:** Raihan Kabir, Yutaka Watanobe, Md Rashedul Islam, Keitaro Naruse, Md Mostafizer Rahman

**Affiliations:** 1 Department of Computer Science and Engineering, University of Aizu, Aizu-Wakamatsu 965-8580, Japan; raihan.kabir.cse@gmail.com (R.K.); naruse@u-aizu.ac.jp (K.N.); mostafiz26@gmail.com (M.M.R.); 2 Department of Computer Science and Engineering, University of Asia Pacific, Dhaka 1216, Bangladesh; rashed.cse@gmail.com

**Keywords:** one-class support vector machine, unknown object detection, cloud–robot system, incremental learning, object segmentation, MobileNet V3, Node-RED

## Abstract

Inter-robot communication and high computational power are challenging issues for deploying indoor mobile robot applications with sensor data processing. Thus, this paper presents an efficient cloud-based multirobot framework with inter-robot communication and high computational power to deploy autonomous mobile robots for indoor applications. Deployment of usable indoor service robots requires uninterrupted movement and enhanced robot vision with a robust classification of objects and obstacles using vision sensor data in the indoor environment. However, state-of-the-art methods face degraded indoor object and obstacle recognition for multiobject vision frames and unknown objects in complex and dynamic environments. From these points of view, this paper proposes a new object segmentation model to separate objects from a multiobject robotic view-frame. In addition, we present a support vector data description (SVDD)-based one-class support vector machine for detecting unknown objects in an outlier detection fashion for the classification model. A cloud-based convolutional neural network (CNN) model with a SoftMax classifier is used for training and identification of objects in the environment, and an incremental learning method is introduced for adding unknown objects to the robot knowledge. A cloud–robot architecture is implemented using a Node-RED environment to validate the proposed model. A benchmarked object image dataset from an open resource repository and images captured from the lab environment were used to train the models. The proposed model showed good object detection and identification results. The performance of the model was compared with three state-of-the-art models and was found to outperform them. Moreover, the usability of the proposed system was enhanced by the unknown object detection, incremental learning, and cloud-based framework.

## 1. Introduction

**Figure 1:**
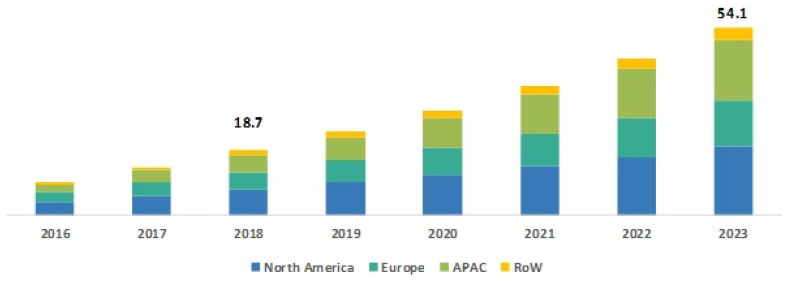
Growth of mobile robot market by region (billion USD). Reprinted with permission from ref. [2]. Copyright 2021 MarketsandMarkets Research Private Ltd.

Currently, scientific research on mobile robots is expanding rapidly. These robots help humans in many application areas and make our daily work easier. Due to their flexibility, such as moving in an indoor or outdoor place autonomously and performing their target tasks, these robots can perform many tasks in the areas of transportation, personal services, construction, medical care, patrolling, museum guides, emergency rescue industrial automation, petrochemical applications, intervention in extreme environments, reconnaissance, operations, planetary exploration, entertainment, and surveillance. Furthermore, various industrial and nonindustrial work is done by robots. Most of this work is done autonomously without any human assistance, which is the biggest advantage of these mobile robots [1]. Actually, we can call a robot autonomous only when it can make its own decisions while performing its tasks. The assigned tasks are properly done with the help of the assistant software and control unit that are part of the mobile robot. Due to these extraordinary benefits, the uses of mobile robots are increasing rapidly at multiple rates. The statistics on the growth of the mobile robot market show that the expected size of this market by the year 2023 will be USD 54.1 billion, where it was USD 18.7 billion in the year 2018, with a compound annual growth rate of 23.71% [2]. Four major information sources, namely current market, parent market, peer market, and exhaustive secondary research on previous market information, were considered in this study to estimate the mobile robot market size (Figure 1).

Automation, high adaptation capability, the demand for reducing human workload, and adoption for personal use are the driving factors of mobile robot market growth. The major mobile robot suppliers are iRobot (US), Honda Motor (Japan), Samsung Electronics (South Korea), LEGO (Denmark), KUKA (Germany), DJI (China), and other mobile robot-making giants. Another study disclosed by the International Federation of Robotics shows that from 2016 to 2019 the sale of domestic service robots increased by 42 million [3].

A cloud-based system provides remote access to computer system resources, high computational power, and especially high data storage capacity (cloud storage). Currently, demand for cloud-based systems is very high because of the advancement of the Internet of Things. Mobile robots need high computational power and large data storage capacity to skillfully perform a large number of operations. Additionally, they need a multirobot communication ability for knowledge sharing and a better understanding of their indoor environments. However, providing these kinds of features to all the robots in a working environment and managing all of them separately is a very costly and difficult task. Therefore, a cloud-based system can make the mobile robot more efficient by storing the robot’s data and sharing the optimal data with other robots after analyzing them. Communication and knowledge sharing are efficient ways to reduce robot learning time and storage space usage [4]. Such a system can establish efficient multirobot communication. These features of cloud-based systems also make mobile robots more autonomous and dynamic. To achieve these advantages, we developed a cloud-based architecture that can efficiently utilize robot data and share optimized knowledge among mobile robots.

Robot localization is one of the most fundamental requirements for a mobile robot in an indoor or outdoor environment. Localization is required for providing services and making decisions on future actions. When operating in indoor environments, localization becomes a challenging task due to the presence of undesired phenomena, such as environmental obstacles, attenuation, and multipath propagation. In the past decade, localization has received a great deal of attention from several researcher groups, who devoted a large amount of effort to this topic. Panchpor et al. surveyed different methods for mobile robot localization for indoor dynamic environments [5]. They presented a simultaneous localization and mapping-based architecture for localization of dynamic environments. They also reported the results of different algorithms, their error rates, and execution times. These algorithms are for different localization problems, such as obstacle avoidance and path planning. In another study, Fascista et al. investigated a localization problem for estimating the position in a dynamic multipath environment using the maximum likelihood approach [6]. They reduced the algorithmic complexity using this approach and also presented an angle-of-arrival-based mechanism for achieving a given trial position under multipath conditions.

To perform services in an indoor or outdoor environment, an expert or intelligent system is important for these mobile robots to obtain a better understanding of the surrounding environment. Additionally, these robots need to recognize surrounding objects and obstacles so that they can make decisions autonomously and instantly for performing different actions. It is important to increase the usability of robot camera viewpoints by recognizing different objects from robot viewpoints. If we can increase the effectiveness of the robot vision system, this will be very useful for helping mobile robots to have a better understanding of the surrounding environment [7]. This is because when these robots need to move from one place to another and can detect all the objects from their viewpoint simultaneously, they can autonomously move without any collisions. For example, if the robot finds a person in its viewpoint, it can make a gesture or avoid collision with that person. Efficient object segmentation can be helpful for efficient object detection [8]. However, many studies have been done without considering the segmentation of multiobject robot vision, which may degrade object recognition performance. Thus, this paper introduced efficient object segmentation for separating multiple objects from captured images. The efficient segmentation of objects from an image may help to detect and classify objects, as well as decrease the computation time, by avoiding the train of complex networks and increasing the performance during mobile robot operation. Also, this segmentation helps to separate objects for unknown object detection.

The classification process comes after the segmentation process is done. After the segmented objects are detected, a problem arises for unknown objects. If the CNN model tries to classify unknown objects, it misclassifies such objects and gives wrong information to the mobile robot, which makes the robot perform an inappropriate action [9]. To overcome this problem, incremental learning can incrementally add the unknown objects in the known list by training the CNN model repeatedly. From this point of view, our goal is to develop an incremental learning method using a one-class support vector machine (SVM)-based outlier detection model by the support vector data description (SVDD) method [10]. In this learning process, first, a one-class SVM separates the unknown objects as outliers, and then these separated objects are learned again and incrementally added to the trained CNN model. Incremental learning is a helpful and important task for an object detection model in terms of detecting new objects and increasing performance [11].

A combination of increasing usability of the robot vision system by recognizing different objects from the surrounding environment, incremental learning for retraining for unknown objects, and a cloud-based system for huge computation power, large data storage, and multirobot communication will make the whole mobile robot system autonomous. To address the above challenges, the main goal of this study was to develop an autonomous multirobot system by incorporating a new unsupervised object segmentation model and a one-class SVM-based incremental learning method for unknown objects. Additionally, a cloud–robot communication architecture is presented to efficiently deploy inter-robot communication and support high computational power and storage capacity overhead. A cloud-based station, including object segmentation, unknown object recognition with incremental learning, and a cloud-based CNN model, is used to analyze the robot data and generate actions for each individual mobile robot in the multirobot system.

The remainder of this paper is organized as follows. Section 2 presents related work, while Section 3 presents details of the proposed model. Section 4 presents and discusses the experimental results, and Section 5 concludes the paper.

## 2. Related Work

Cloud robotics, object segmentation/detection, and incremental learning using different image-processing and machine-learning techniques are very popular research topics. Several researchers have investigated these topics to enhance the environmental knowledge of mobile robots and manage multirobot systems. However, their performance is suboptimal and much room remains open for performance enhancement by integrating incremental learning with an unsupervised object segmentation technique and a cloud-based multimobile robot system. Thus, the main purpose is to propose an organized and efficient model to achieve this research goal. Table 1 summarizes the related works we reviewed according to the methods used, their advantages, and their drawbacks.

In 2010, the idea of cloud robotics was first proposed by Kuffner, which greatly increased the robots’ operating capability [12]. This concept is accomplished by a remote server’s heavy-CPU task offload capability and sharing of cloud computing resources with the robots. Thus, these robots can use cloud-based services to extend their operating capability. In different sectors of industry, the need for robots has rapidly increased. In the manufacturing sector, such as assembly/disassembly, industrial cloud robotics is a well-known topic. For this type of industrial application, energy analysis, path simulation, production process control capability and synchronization, real-time operating capability, and local robotic stability are the major critical issues. By using cloud robotics, critical issues and the conflict between the requirement of local robot control and the need for heavy computational power can be solved [13,14].

Independently, Liu et al. proposed a novel cloud platform that can be used for service robots. They designed a platform, named the cloud robotics intelligent cloud platform [15], comprising an interface layer, algorithm layer, service pool, and gateway layer. The gateway layer controls the robot accessibility, while the interface layer controls different types of protocol, such as the management protocol, motoring protocol, service request protocol, and others. The service pool has several data analysis-based services. In their method, different kinds of machine-learning algorithms can be implemented in the algorithm layer. They experimented with their proposed method using a voice recognition service and a scaling test of their cloud service. To do that, they used a service-oriented architecture and a local framework.

In another study, Watanobe et al. worked in the field of data acquisition in cloud robotics [16]. The main focus was on the need for different software components for cloud robotics. These software components need to acquire different kinds of data from heterogeneous devices. However, to create these components and their corresponding services, various tasks need to be performed. From this point of view, they proposed a theory for a cloud-based data acquisition framework and then presented a concrete implementation for their architecture and framework. In this framework, they created an opportunity for developers to define components and objects by simply creating scenarios in an ontology language. This process will enable the functionality of data acquisition in a cloud-based system by automatically generating the required software components.

Meanwhile, Chen et al. gave a conceptual view of cloud robotics and its nature [17]. Their paper presented the cooperation frameworks of robotic physical tasking, knowledge sharing, and computational tasking. Also, they described the possibilities, benefits, and challenges of the three proposed frameworks. In another paper, they described network cloud robotics, quality of service, and an allocation algorithm based on robot streaming workflow. They also discussed the cost, energy efficiency, and optimization latency for cloud robotics [18]. 

In yet another study, Badawy et al. proposed a human mobile robot architecture based on cloud computing, where real-time constraints can be overcome by mobile robots [19]. They proposed a CNN model using a gas algorithm, and they achieved a 25–45% response time improvement. Rahman et al. presented a task-offloading method for a multirobot system. They used a cloud for their offloading system and applied it to task distribution for warehouse sorting. They described it as cloud-based energy-efficient offloading [20]. 

In the computer-vision and image-processing area, the meaning of image segmentation is to separate the input image into different meaningful segments that can be analyzed easily. Al-Hafiz et al. presented a Canny edge detector and threshold-based red-blood-cell segmentation algorithm [21]. They applied this to detect the boundary of a threshold image and obtained an 87.9% segmentation ratio from the bioimage. Espinace et al. presented an indoor scene identification technique where they tried to extract low-level features from different indoor objects for classifiers [22]. They also tried to obtain structural and geometrical information using a 3D range sensor to increase the performance of their proposed model.

Separately, Wang et al. presented a survey of image-segmentation methods and their evaluation [23]. They summarized different supervised and unsupervised image-segmentation methods and their evaluation quality. They also experimented with different algorithms for medical, natural, and other related images, and ranked the algorithms based on their effectiveness. 

In the machine-learning field, incremental learning is an efficient method that helps to extend the knowledge of existing trained models by repeatedly retraining the model with data that become available over time. This increases the model performance and detection ability. Chen et al. presented a hybrid incremental-learning method that can detect new objects and increase the recognition quality of the model [24]. They used an SVM in their model to make it an incremental learning model. They utilized a subset of the ImageNet dataset and conducted a hand-held object recognition experiment to evaluate their proposed model. They compared the results for different aspects and demonstrated that it had a good performance.

In the machine-learning sector, a one-class SVM is a state-of-the-art method for abnormality or outlier detection. Amer et al. proposed a method for unsupervised anomaly detection by enhancing the one-class SVM [10]. They modified the SVM for better unsupervised abnormality detection. Two modifications were applied: an eta one-class SVM and a robust one-class SVM. The benefit of these modifications was that normal data contribute more than the outliers. They experimented with UCI machine-learning repository datasets and compared them with other algorithms. The comparison showed that the eta one-class SVM performed better than all other algorithms they considered.

Separately, Mourão-Miranda et al. proposed a one-class SVM-based outlier detection application for depressed patients’ facial expressions in fMRI images [25]. Their model detected healthy and unhealthy patterns using outlier detection. They also extracted brain anatomical features and established a relationship between the patients’ scores on the Hamilton rating scale for depression and prediction by one-class SVM. Based on the detected outliers, they obtained results that predicted which patients would require treatment in the future. For all the patients, one-class SVM predicted 89% of nonoutliers and 52% of outliers.

Currently, system-on-a-chip technologies are growing rapidly. Along with this, the demand for lightweight intelligent-vision systems for low-power microcomputers is increasing. With that point in mind, researchers are trying to make CNN models more compact, with low computational complexity and small model size, while maintaining standard model performance. MobileNetV3 is one of the lightweight state-of-the-art CNN models. Glegoła et al. proposed a CNN model named MobilrNetV3-Small-Pi [26]. In their study, they first ran various lightweight CNN models on a raspberry pi-4. They then modified the MobileNetV3 and proposed MobileNetV3-Small-Pi for better hardware utilization. They performed experiments using their proposed architecture and increased the execution speed. Moreover, Prasad et al. studied MobileNetV3, which is a resource-constrained platform [27], and proposed a further size reduction of a modified MobileNetV3 architecture while maintaining almost the same classification performance. Their proposed modified model size was 2.3 MB, whereas the main MobileNetV3 model size was 15.3 MB [28]. They experimented with their model on the CIFAR-10 dataset and achieved a performance of 89.13%, whereas the main model performance was 88.93%.

Meanwhile, Howard et al. and Nan et al. experimented on the FERPlus and RAF-DB datasets using an enhanced lightweight MobileNet model named A-MobileNet [29]. They enhanced the performance by introducing an attention module into the MobileNet-V1, as well as combining the SoftMax loss and center loss. This process makes an interclass distance increment and an intraclass distance decrement and adjusts the model parameters. This MobileNet model achieved very good performance compared to others.

**Table 1:** Summary of state-of-the-art methods, including their advantages and drawbacks.

Reference	Method	Advantages	Drawbacks
[15]	Cloud robotics intelligent cloud platform (CRICP)	Proposed a cloud platform for service robots with different service layers. These layers help to solve different problems such as access control, protocol injection, algorithm implementation, and others.	Did not provide much information on how they physically implemented the cloud services and how the service robots communicate with the cloud platform.
[17]	Robotic cooperation in cloud robotics	Presented a conceptual view and framework of robotic cooperation for cloud robotics such as robotic physical and computational task cooperation, knowledge-sharing cooperation.	Provided only conceptual models and did not provide much information on how the databases are managed.
[19]	Image clustering algorithm and growing neural gas algorithm	A human–cloud–robot architecture and a data-flow mechanism for the robot vision system.	Did not adopt any incremental learning method, which helps to enhance the robot’s vision capability.
[24]	Incremental learning for hand-held object recognition	Hybrid incremental learning method for the new data and classes.	There were no efficient unknown data separations and segmentations, for efficient incremental learning
[25]	One-class SVM	This method was used as an outlier detection application for depressed patients’ facial expressions of fMRI response in addition to the extracted brain anatomical regions feature, and achieved 52% of accuracy to detect patients as outliers.	No incremental learning approach was adopted, for better outline detection performance.
[27,29]	MobileNet	Presented a modified MobileNet model for lightweight devices based on MobileNetV3 and MobileNetV1.	Did not use any efficient segmentation method, so that multiple objects can be detected from an image.

## 3. Proposed Model

**Figure 2:**
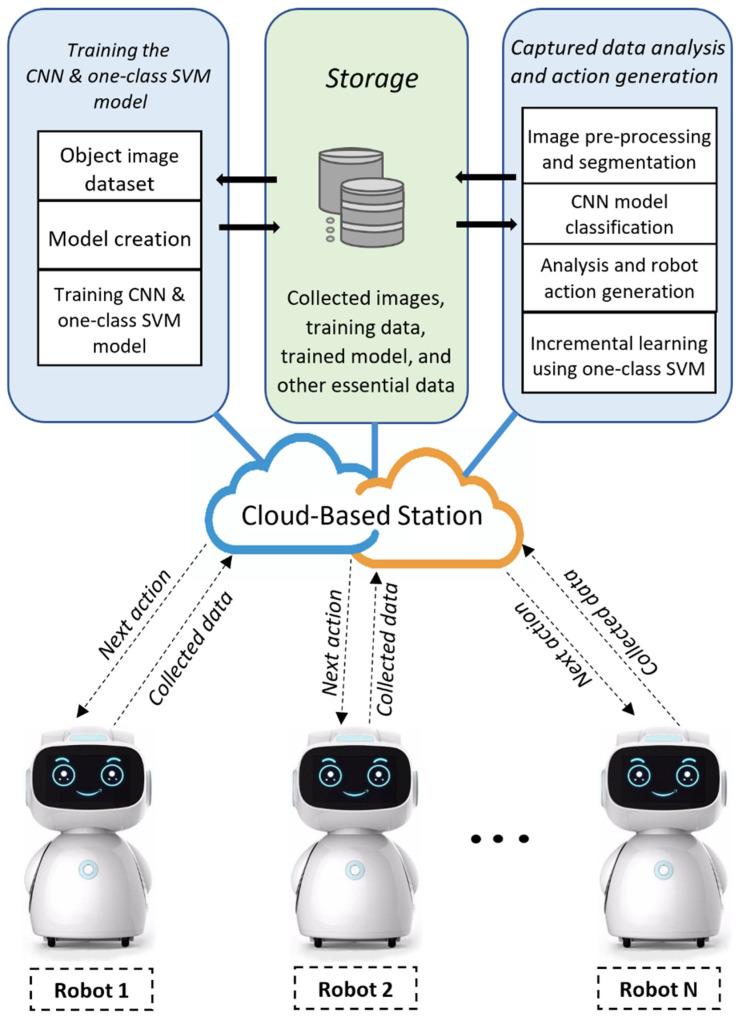
General block diagram of the proposed cloud–robot system.

Figure 2 presents the general framework of the cloud-based mobile robot system. The proposed framework consists of two major parts: a robot local station and a cloud-based station. All the main operations (such as image preprocessing, object segmentation, object detection, CNN model training/execution, object classification, and incremental learning using one-class SVM) are performed by the cloud-based station, and all the robot data (such as camera image data, the trained model, robot sensor data, training data, and other necessary robot data) are stored in the database of this cloud-based station. Robot actions are also generated in this station and then sent to the individual robots.

The robot local station collects camera image data from its surrounding viewpoint and then sends these data to the cloud-based station for analysis. On the cloud-based station, image-processing and machine-learning algorithms process these data and generate actions for the robots. These actions are performed by an onboard controller, the robot operating system, motor drivers, and other actuators. 

Furthermore, multiple robots can be controlled using the model because each robot connected to the cloud-based station shares its data and communicates with other robots (e.g., sharing location and knowledge). Also, all the mobile robots store their data in the central cloud storage and receive their next action from it using their individual robot identity (ID) number.

### 3.1. Robot Data Analysis Process in Cloud-Base Station

**Figure 3:**
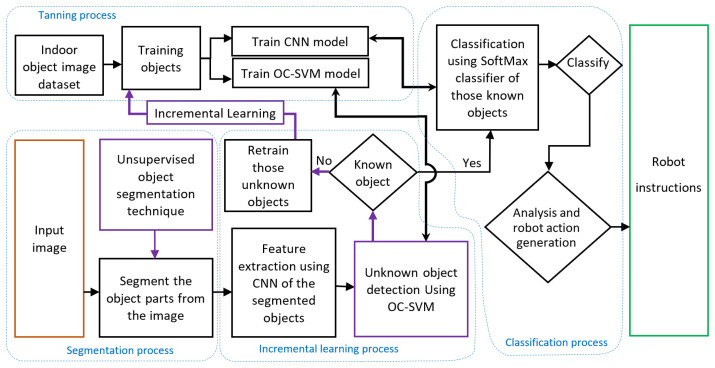
Robot data analysis process in the cloud-based station.

Figure 3 presents the flow of the robot data analysis process, which is implemented on the cloud-based station. In this block diagram, the data analysis process has five parts: (a) training process, (b) segmentation process, (c) incremental learning process, (d) classification process, and (e) robot instruction. These processes are performed using different machine-learning/deep-learning and image-processing techniques.

In the training process, an object image dataset is used to train the CNN and one-class SVM model. The trained model is then stored in the database of the cloud-based station for the purpose of unknown object detection and known object classification. 

In the segmentation process, the input images come from the robots and these images are segmented using an unsupervised segmentation algorithm. This allows us to obtain all objects from an image and process the segmented objects for recognizing environmental objects and obstacles.

In the classification process, objects are classified using a classifier. In our model, the SoftMax classifier is used for classification purposes. After classification, the object information is used for robot action generation, such as moving forward and backward or reacting with a gesture or other such actions. However, some objects may not be learned in advance by the robot. Thus, a one-class SVM is implemented to detect unknown objects and to implement the incremental learning process.

In the incremental learning process, features of segmented objects are first extracted using a CNN model, and are then used to detect unknown objects. Trained one-class SVM models separate the unknown objects based on these extracted features. Unknown objects help to train the CNN and one-class SVM models again. If the object is known, then it will enter into the classification process. The entire system incrementally learns about new objects using this incremental learning process.

Furthermore, in the robot instruction part, the generated action helps to formulate robot actuator instructions. For example, if the robot is wheel-based, then the instruction includes how much the wheel needs to rotate in each direction; if the robot has arms for making a gesture, then the instruction includes how much each arm joint needs to move. These robot instructions are then sent to the individual robots from the cloud-based station using the robot ID.

### 3.2. Unsupervised Segmentation Method

Generally, an image with a dynamic and random background is very complex, and objects are difficult to segment into parts [30]. Additionally, without proper segmentation, classification of every object in an image is difficult and existing segmentation algorithms are suboptimal, are complex, and have a high time complexity. Therefore, we propose a new unsupervised segmentation method, which is less complex and has a low time with good performance.

In this segmentation method, different lightweight and less complex image-processing algorithms are used for segmenting an image containing multiple objects. This segmentation algorithm comprises Canny edge detection, Otsu’s thresholding, Floodfill, and BoundingBox image processing.

**Figure 4:**
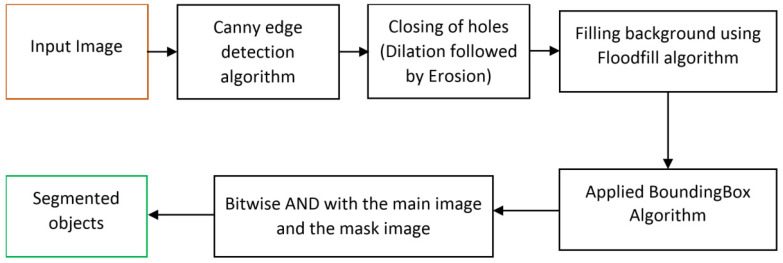
Proposed unsupervised segmentation method flow diagram.

Figure 4 shows the flow diagram for the proposed unsupervised segmentation algorithm. This method contains several steps.

Step 1 Obtain the input image and apply Canny edge detection and Otsu’s thresholding algorithm, which detects the object edges [31]. By fine-tuning the edges and closing the noise holes, the actual object edges are constructed from the RGB input image.
Step 2 Apply the enhanced Floodfill algorithm to fill in the background, and separate the foreground and background from the edged image obtained from step 1.
Step 3 Apply the BoundingBox algorithm to obtain the boundary mask for the object parts.
Step 4 Finally, apply a bitwise AND operation between the actual input image and the masked image, which separates the object parts from the input RGB image.

### 3.3. One-Class Support Vector Machine Using SVDD

A one-class SVM is used for outlier detection and constituting an incremental learning process in the proposed model. This is an SVM modified to be a single-class learner that tries to find a hyper-sphere among the instances of the normal classes [32]. Because this method judges new data as normal or abnormal, all observations inside the hypersphere are normal and those outside the hypersphere are abnormal or outliers. The user-defined regularization parameter *C*, radius R, and center location of the data a strongly influence the one-class SVM’s performance. In Equation (1), an optimization method helps to obtain the values of R and a in a dataset containing N  data points, $\left\{x_{i},\;i=1,\ldots ,N\right\}$. However, class data are explored for determining the value of the parameter *C* by calculating the fraction of outliers (v).

(1) $$F{\left(R,a\right)}_{\min}=R^{2}+C\overset{N}{\underset{i=1}{\sum }}\xi_{i};C=1/Nv,\;\mathrm{subject}\;\mathrm{to}\;\|x_{i}-a{\|}^{2}\leq R^{2}+\xi_{i};and\;\xi_{i}\geq 0;\;\;\forall i=1,\ldots ,N.$$

Here, $\xi_{i}$ is a slack variable that controls the allowed number of misclassifications in the SVDD. Also, in Equation (1) the Lagrangian dual formulation L(α) is rebuilt in addition to a kernel function as a solution for the optimization problem, and the equation is given as:

(2) $$L{\left(\alpha \right)}_{\min}=\overset{N}{\underset{i=1}{\sum }}\alpha_{i}\Phi \left(x_{i}\cdot x_{i}\right)-\overset{N}{\underset{i,j=1}{\sum }}\alpha_{i}\alpha_{j}\Phi \left(x_{i}\cdot x_{j}\right),\;\mathrm{subject}\;\mathrm{to}\;0\leq \alpha_{i}\leq C\;\;\;\;\forall i;\;and\;\sum_{i=1}^{m}\alpha_{i}=1.$$

Here, Φ is a Gaussian kernel function, $\alpha_{i}$ represents Lagrange multipliers, and Φ is expressed as:

(3) $$\Phi \left(x_{i}\cdot x_{j}\right)=exp\left(\frac{-\|x_{i}-x_{j}\|{}^{2}}{\sigma^{2}}\right).$$

In the Gaussian kernel function, the plasticity of the hypersphere and the width of the kernel are controlled by the parameter σ. To obtain optimal performance from the one-class SVM, appropriate user-defined values for σ and v need to be carefully chosen.

### 3.4. Feature Extraction Using CNN and Classifier

Feature extraction for the mobile robot’s image data is very important for the robot vision system and object detection. The accuracy of this kind of vision system mostly depends on extracting good features to allow a robot to differentiate between different kinds of objects in front of it, such as a person or a dog. Our proposed model uses MobileNet [28], which is an efficient and lightweight CNN architecture. It was constructed for embedded and mobile vision systems. It follows a pointwise convolution for performing a depth-wise separable convolution by splitting the convolution, which makes this CNN architecture a very lightweight network. The basic MobileNet architecture follows multiple MobileNet blocks, including convolution (Conv) blocks, rectified linear unit (ReLU) blocks, and BatchNormalization blocks. In this architecture, the number of strides (s1, 2) from the depth-wise convolution layer (Conv dw) is followed by the number of strides from the convolution layer (Conv), which means Conv dw/s1 followed by Conv/s1. The number of filters and the kernel size are determined by the “filter shape.” Also, the number of filters increases in a linearly incremental manner from 32 to 1024 (i.e., 32 to 64, 64 to 128, then 256, and so on). The input image size is 224 × 224 × 3, and for deeper network layers, the input size decreases while its dimensions increase. At the end of the network, the SoftMax classifier assigns the image to 1 of 1000 classes. Also, two very simple hyperparameters provided by this architecture help to build small low-latency models, which are very lightweight. Among these hyperparameters, one is a width multiplier that helps to reduce the number of channels and another one is a resolution multiplier. The spatial dimensions of feature maps are reduced using the resolution multiplier hyperparameter.

**Figure 5:**
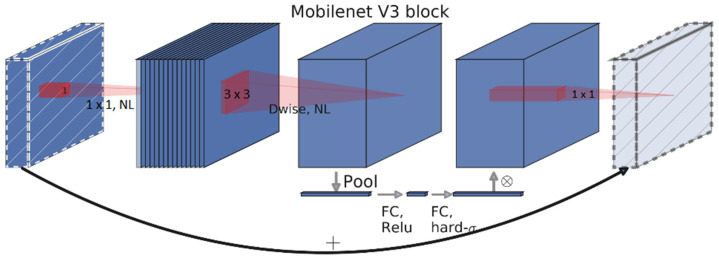
Building blocks of MobileNet V3 architecture.

MobileNet V3 is an improved version of MobileNet V2, and it consists of squeeze (derived from squeezeNet) and excite blocks (derived from excitation networks), as well as inverted residual bottlenecks derived from MobileNet V2 [29]. Figure 5 shows the building blocks for the MobileNet V3 architecture. MobileNet V2 is also an enhanced form of MobileNet V1. In terms of speed and accuracy, this enhancement more than doubles the performance. The MobileNet V3 network architecture comprises a 1 × 1 projection layer, a pointwise 1 × 1 expansion layer, and a depth-wise 5 × 5 or 3 × 3 convolution layer. Squeeze-and-excite blocks are added to highlight the neurons that contribute to network performance.

### 3.5. Incremental Learning Method

Our incremental learning method is based on a one-class SVM. A trained one-class SVM can detect an object that is known or unknown by the model, but the limitation here is that it can only detect a single class or one kind of object at a time. To overcome this limitation, we developed a model that can detect multiple classes or multiple kinds of objects. For example, if we have trained class set A = (X_1_, X_2_, X_3_, …, X_n_) and untrained class set B = (Y_1_, Y_2_, Y_3_, …, Y_n_), then the model can detect all these classes in set A as known objects and classes in set B as unknown objects. If the objects are unknown, then the model will incrementally train the model for these unknown objects, and known objects are classified separately.

**Figure 6:**
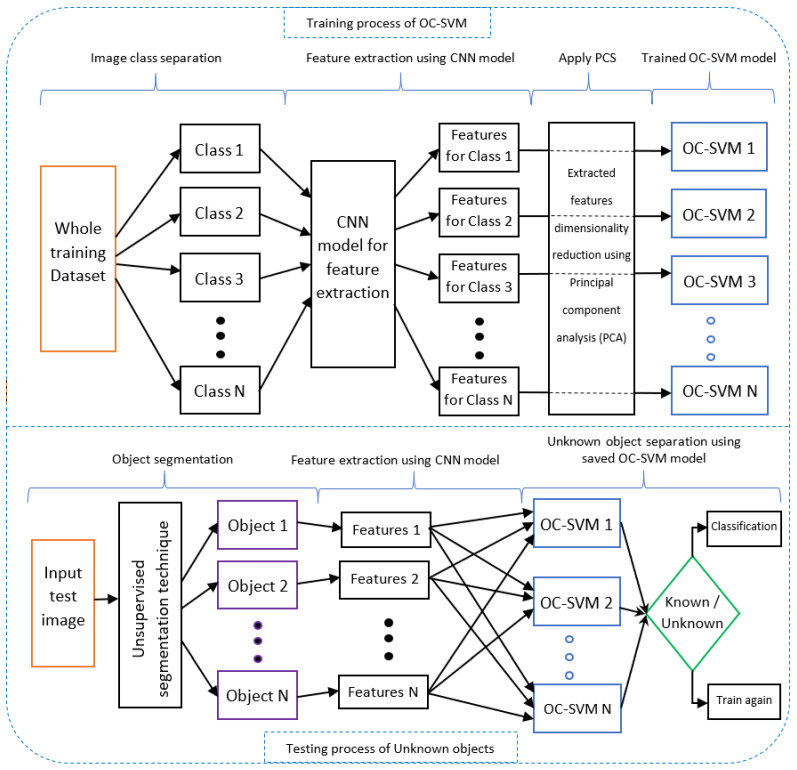
Block diagram of the training and testing process for the proposed incremental learning method.

The proposed incremental learning method in Figure 6 has two parts: the training process for one-class SVM models and the testing process for known and unknown objects. First, in the training process, all the classes (1-N) are separated, and then a CNN model extracts the features of these classes. A principal component analysis (PCA) is applied, which reduces the dimensionality of the feature vectors. This dimensionality reduction helps the one-class SVM to be trained properly. This is because a CNN model extracts thousands of features from the images, but dimensionality reduction of these features allows the one-class SVM model to be trained quickly. Meanwhile, after the PCA, these features are used to train the one-class SVM. Here, features of each class (1-N) train and create a one-class SVM trained model for each class (1-N).

In the testing process, at first, the input image is segmented into different objects using an unsupervised segmentation technique. These object features are extracted using the same CNN model that is used in the training process. The PCA is applied to reduce feature dimensionality, and the features of each object help to separate known and unknown objects. If the objects are known, then they will enter into the classification process; otherwise unknown objects are learned again incrementally. This incremental learning process is automated and makes the learning process more efficient.

### 3.6. Cloud-Based Station and Different Layers

**Figure 7:**
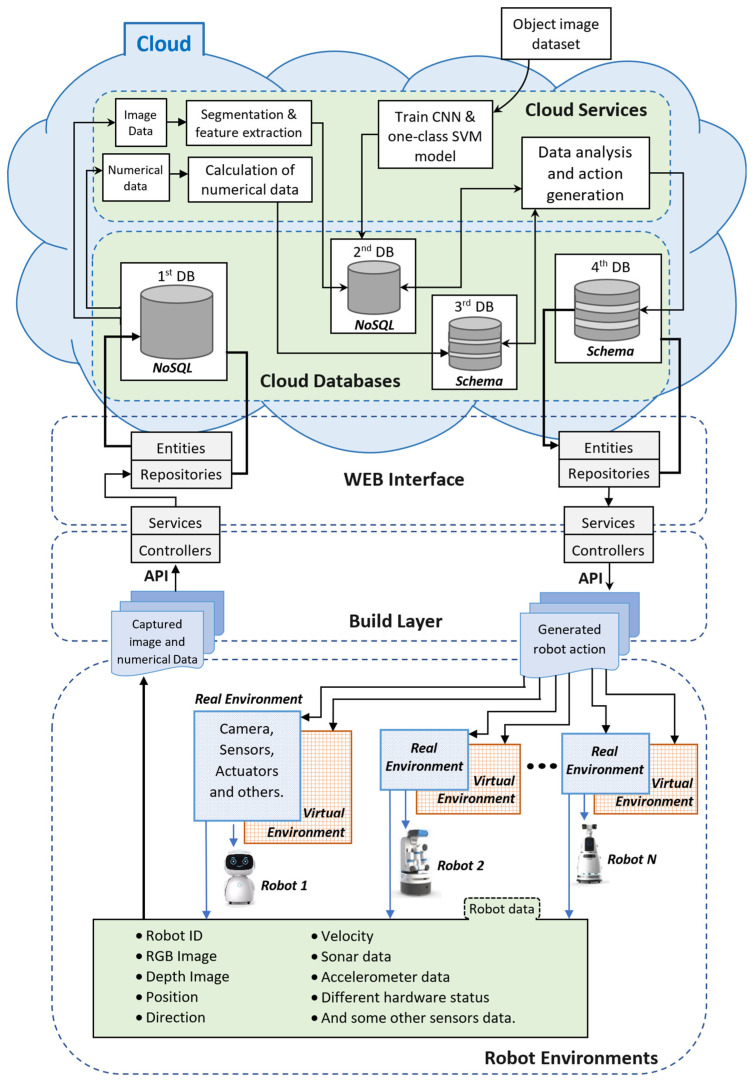
Block diagram of proposed cloud-based architecture.

A cloud-based station for a multirobot system is a very useful and efficient way to control robots by managing and analyzing their collected data. Figure 7 presents the proposed cloud-based architecture, where the cloud-based station analyzes and manages the robot data as well as generates the robot instructions [14].

This architecture has five interconnected components: (a) robot environment, (b) build layer, (c) web interface, (d) cloud database, and (e) cloud services. The cloud-based station includes components (c), (d), and (e).

The robot environment is subdivided into real and virtual environments. The real environment helps to collect and present different robot data, whereas the virtual environment helps to manipulate the robots. These robot environments send the robot data to the cloud-based station with the help of APIs in the build and web interface layer (including services, controllers, entities, and repositories) using the robot ID number. Requests between robot local and cloud-based stations are managed by the controllers, and a bridge to the corresponding database is made.

In the cloud database, there are four multistage databases; some are NoSQL databases, and some are relational databases. These databases help to store and manage raw and analyzed data. The cloud service part has several algorithms that run on the cloud-based station for robot data analysis. This cloud-based architecture analyzes the data from several robots at a time, meaning that multiple robots can be controlled and manipulated by this architecture.

## 4. Experimental Results and Discussion

This section presents the implementation, experimental results, and discussion for our proposed model. First, we explain the implementation of the cloud-based architecture, and then show results for the unsupervised segmentation algorithm. The one-class SVM and incremental learning algorithm implementation are then described. Finally, we describe the entire proposed model by combining all the implementations, and we show the overall object and obstacle classification results.

### 4.1. Implementation of Cloud-Based Architecture

This section describes the implementation of the proposed cloud-based station. The architecture contains two basic stations: the robot local station and the cloud-based station. We implemented the robot local station on four raspberry-pi 4B microcomputers and we used a powerful computer as a cloud-based station. The station configuration was one Intel^®^ Core™ i9 CPU with 3.60 GHz and 64 GB RAM, and one NVIDIA GeForce RTX 3090 GPU. The computer has 10 physical cores with 20 logical processors for multitasking. We implemented the entire system in a Node.js-based environment called Node-RED. This was originally developed by IBM and is a flow-based graphical programming user interface. There are various types of nodes where we have written and built our code in JavaScript and python for physically implementing the proposed architecture. For storing the data in the cloud-based station, we used MongoDB as a NoSQL database and MySQL as a relational database.

**Figure 8:**
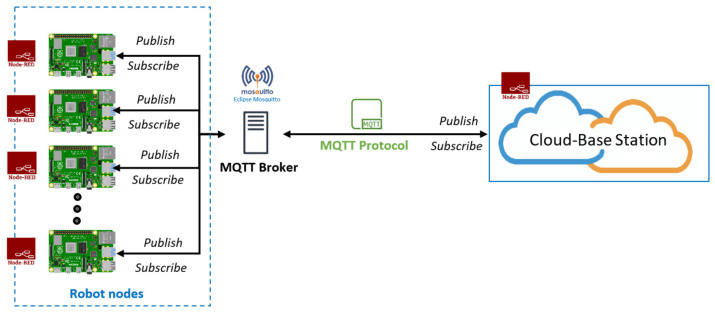
Communication between multiple robot nodes and cloud-based station using MQTT network protocol.

For communication, we used the MQ Telemetry Transport (MQTT) network protocol, which is a publish/subscribe-based lightweight network protocol that follows the TCP/IP model. It provides bi-directional, lossless, and ordered communication. Communication through the MQTT network protocol between the cloud and robot nodes requires a broker server. For the broker server, “Eclipse Mosquitto” was used, which is an open-source message broker that implements MQTT protocol versions 5.0, 3.1.1, and 3.1. In our implementation, the MQTT protocol version was 3.1.1. Eclipse Mosquitto provides a local MQTT-broker server that is a lightweight and easy-to-use broker server for MQTT networks. For communicating with different robot nodes, there is a unique identity called “Topic,” which helps to communicate with multiple robot nodes. Figure 8 shows a schematic diagram for this communication.

**Figure 9:**
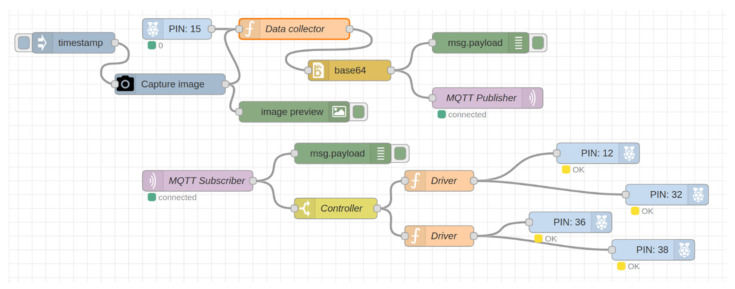
Implemented data flow in the robot local station in the Node-RED environment.

First, the robot local station was implemented on raspberry pi microcomputers as shown in Figure 9. We captured images and sensor data from the raspberry pi camera and sensors. After that, we sent the data through the MQTT publisher node to the cloud station, which performed different operations on those data and sent the results back to the pi using the MQTT subscriber node to control a wheel-based robot.

**Figure 10:**
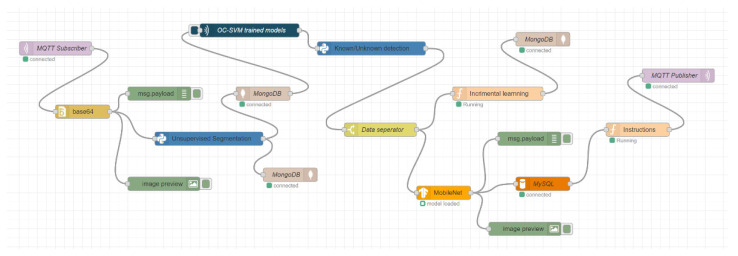
Implemented data flow in cloud-based station in Node-RED environment.

Figure 10 presents the implementation of the cloud-based station as part of the proposed architecture. In the cloud-based station, the data from the robot local station were received and then the unsupervised segmentation algorithm was applied to segment the images into different object parts. Those segmented objects were stored in a NoSQL database and retrieved for the next operation. Furthermore, we used the trained one-class SVM models for detecting known and unknown objects. In this stage, unknown objects were stored in the NoSQL database for incremental learning by training the model again, while known objects were classified using a trained MobileNet machine-learning model. This classification information was stored in a relational database and was used to help generate the next robot instructions. Finally, the robot instructions were sent to the robot local station by the MQTT publisher node for performing the next robot action.

### 4.2. Dataset

The dataset is the most important part of a machine-learning-based implementation. For that reason, we used a benchmarked large-scale object detection dataset named the MS Common Objects in Context (COCO) dataset for validating the proposed incremental learning, object segmentation, and classification. This COCO dataset is composed of 80 different object classes [33]. It also has 123,287 images and 886,284 instances. The dimensions of the RGB images are 256 × 256 × 3. Among these 80 classes, for the experiments and validation, we selected 12 indoor object classes: person, chair, table, dog, stop sign, cat, bottle, potted plant, couch, TV, microwave, and teddy bear. In this study, images from these classes were used for training; whereas for testing, in addition to the images from the dataset, we used some of our own captured images from the robot camera. 

### 4.3. Unsupervised Object Segmentation

**Figure 11:**
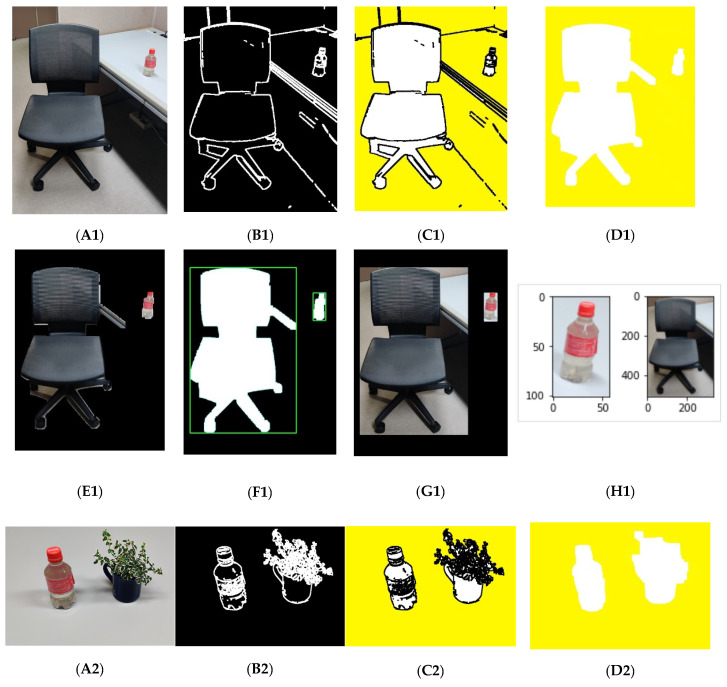

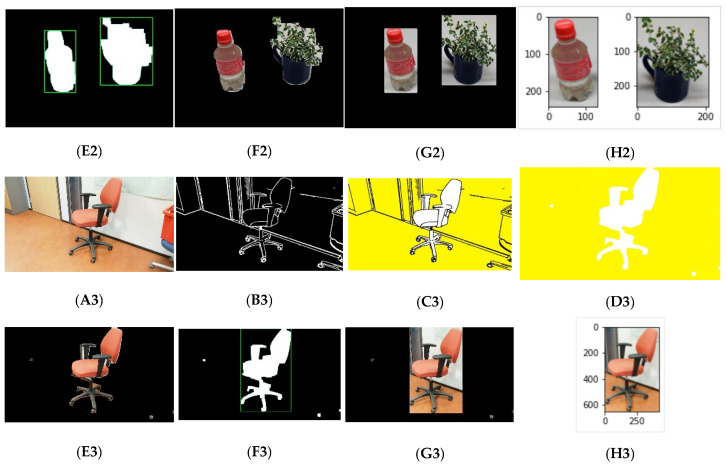
Experimental outcomes of the proposed unsupervised segmentation method. (**A1**–**A3**) Input image, (**B1**–**B3**) detected edges using Canny algorithm, (**C1**–**C3**) inverted image after applying enhanced Floodfill algorithm, (**D1**–**D3**) foreground of Floodfill image, (**E1**–**E3**) foreground part separated from real image using Floodfill image, (**F1**–**F3**) foreground of BoundingBox image, (**G1**–**G3**) Bitwise AND with mask BoundingBox image and main image, and (**H1**–**H3**) all segmented objects.

As described in Section 3.2, efficient segmentation is important because classification of individual and segmented objects from the background may improve the model performance. The proposed unsupervised segmentation can be used to separate multiple objects easily from a dynamic environment. Also, by detecting unknown objects among them and classifying only the known objects, actual object information can be reliably given to the robots to allow them to perform appropriate actions. The proposed unsupervised segmentation algorithm was developed using image-processing algorithms, including Canny edge detection, Otsu’s thresholding, BoundingBox, and the Floodfill algorithm. Within a few steps, as described in Section 3.2, this model can segment different objects from the multiobject images, and Figure 11 shows the segmentation results. First, the Canny edge detection and Otsu’s thresholding detects the edges of the objects in the image, and Figure 11(B1–B3) shows the resulting images, whereas Figure 11(A1–A3) shows the actual RGB images used as the input. After that, an enhanced Floodfill algorithm separates the foreground as shown in Figure 11((C1–C3)–(E1–E3)). Figure 11((F1–F3),(G1–G3)) presents the output of the BoundingBox algorithm. The last masked image helps to separate the objects from the input RGB image as in Figure 11H. These segmented images were used for training and classification.

### 4.4. Implementation of Incremental Learning with One-Class SVM

In our proposed model, a one-class SVM is used to implement the incremental learning method. As described in Section 3.5, first we trained the one-class SVM gradually or incrementally with the object classes using the selected dataset. In this process, 12 different classes were selected and 4 separate test cases were executed. Table 2 represents the results and performance for each test case. In test case 1, we made five separate trained one-class SVM models by training with five classes (person, chair, table, dog, and stop sign). The trained one-class SVM models were then tested with 50 samples from each known class along with 200 samples from unknown classes, and the obtained performance is shown in Table 2. In test case 2, two additional one-class SVM models were prepared from the unknown classes that were detected as unknown in test case 1. Furthermore, the performance of the trained classes was tested in addition to 200 unknown class samples and got the detection performance. Similarly, experiments for test cases 3 and 4 were performed and incrementally added the unknown classes in the trained model list by training with the unknown sample data. By this method, the proposed model incrementally learns new object classes and gives the exact object information to the robots so that they can perform the correct operation. 

**Figure 12:**
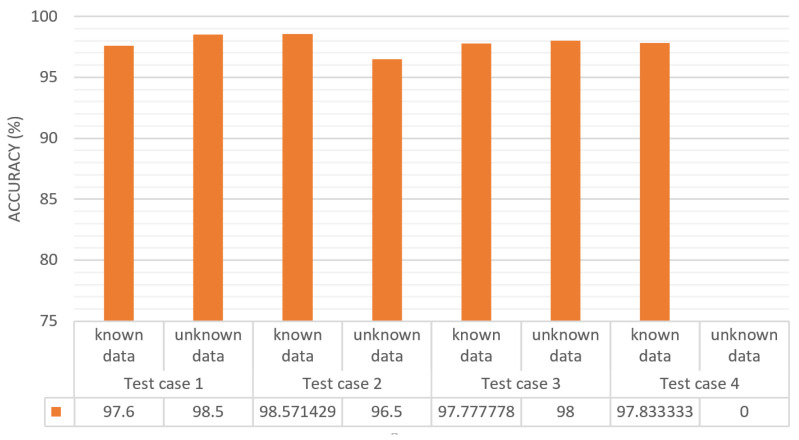
Classification accuracy for known and unknown data for each test case.

Table 3 shows the sample detection outcome for test cases 1 and 2. In a particular test case, an object is unknown if all the trained one-class SVM model outputs are zero (0) and known if one of the trained one-class SVM model outputs is one (1). The unknown object may misguide the detection result. Thus, the unknown objects are trained again. In Table 3, the detection outcomes for person, dog, and chair are one (1) because these classes are already known in test case 1. But the detection outcome for the bottle is zero (0) because it is still unknown and not trained yet. In the same way, in the test case 2, potted plant is unknown and the other classes are known. Figure 12 shows a bar chart representing the classification accuracy for the four test cases (described in Table 2) for the implementation of one-class SVM and incremental learning. In this figure, the *x*-axis represents the test cases and the *y*-axis represents the accuracy of each test case separating known and unknown data, as shown in Table 2. In test case 1, the known data prediction rate is 97.6% and for the unknown data is 98.5%. The results for test cases 2 and 3 are similar to those for test case 1, but in test case 4, the unknown data prediction field is zero because there were no unknown data since all the considered classes were learned.

**Figure 13:**
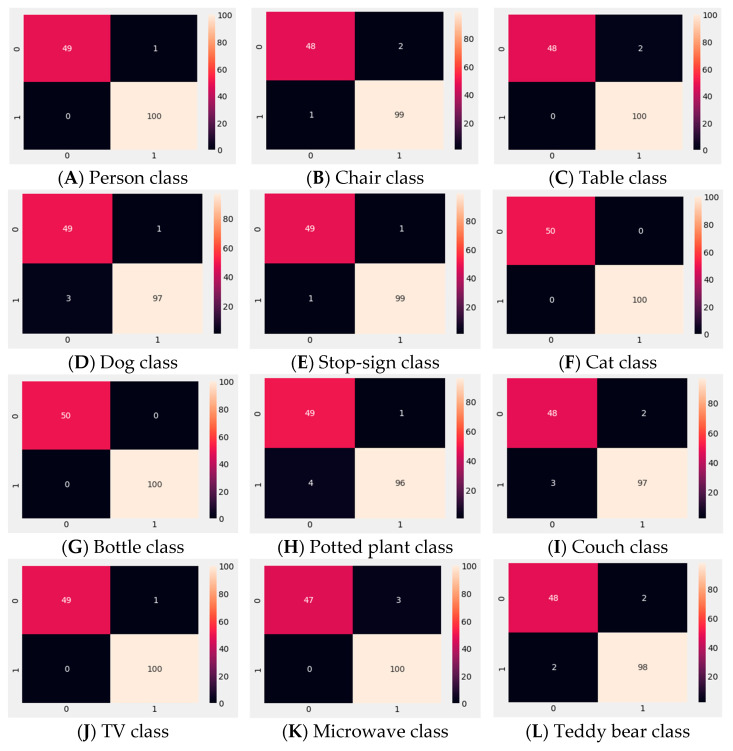
(**A**–**L**) confusion matrix for known and unknown data detection for each class.

To evaluate each trained one-class SVM model’s performance, an experiment with 100 known and 50 unknown data for each class was conducted. In other words, those 100 data were from a particular trained one-class SVM model class and the other 50 data were from other random unknown classes. Figure 13 and Table 4 show the results of this experiment. Figure 13 represents the confusion matrix for known and unknown object detection results for each class.

Table 4 presents the individual class precision, recall, f1-score, support, and accuracy for known and unknown data. It also shows each class performance for the trained one-class SVM models. To calculate the performance, we used the following formulas:

(4) $$Precision\;\left(Pre\right)=\frac{TP}{TP+FP},$$

(5) $$Recall\;\left(Rec\right)=\frac{TP}{TP+FN},$$

(6) $$F1=2\times \frac{Pre\times Rec}{Pre+Rec}.$$

### 4.5. Object and Obstacle Classification

**Figure 14:**
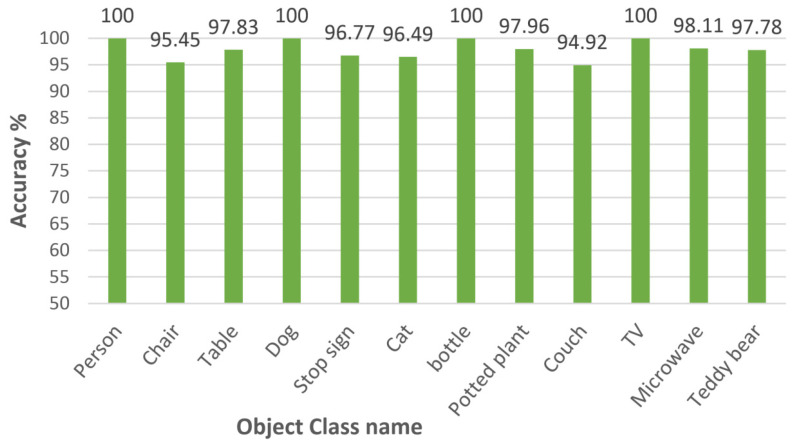
Individual class classification accuracy.

**Figure 15:**
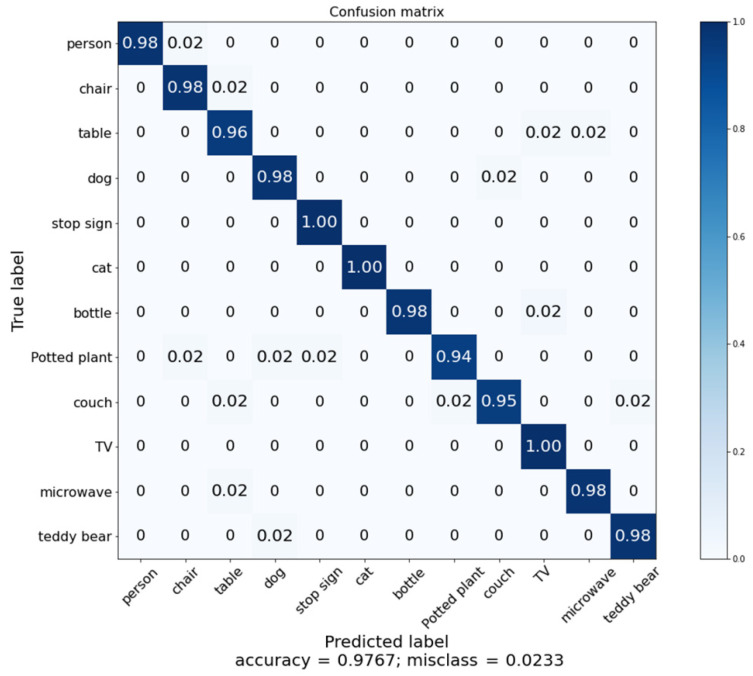
Confusion matrix for classification accuracy.

Our proposed cloud robotics model was tested with some separate experiments, such as an implementation of cloud-based architecture, an unsupervised segmentation method, an implementation of incremental learning with one-class SVM, and classification with the MobileNet model. We conducted the experiments one by one. First, we implemented the proposed unsupervised segmentation method, and then we implemented the incremental learning method with one-class SVM. After that, we implemented the cloud-based architecture using the Node-RED environment, where we added the two implementations and prepared a trained MobileNetV3 model for classification. To evaluate the performance of the proposed model, the proposed method was implemented using the “out” subset of COCO, including the 12 classes described in Section 4.2 for classification purposes. To avoid bias in the dataset for training and testing, the K-fold cross-validation was applied with a *K* value of 5. Every result is an average of *K* iterations. We achieved an overall classification accuracy of 97.67% with our proposed model, which is a good performance. Figure 14 shows the classification accuracy for each class and Figure 15 shows the confusion matrix for that performance. We expect our proposed model to make cloud–robot systems more efficient, robust, and reliable.

**Figure 16:**
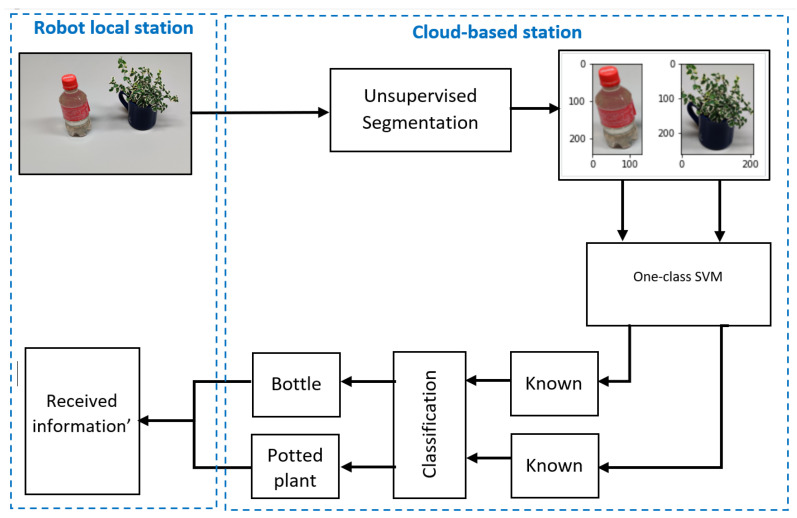
Schematic diagram for a single image in the multirobot experiment.

As described in Section 4.1, four raspberry-pi-4Bs were used for implementing the multirobot system. The data flow of the Node-RED nodes on each robot node is shown in Figure 8 and Figure 9. Using the implemented nodes and flows shown in Figure 9, each robot node sent the collected data and received the resultant data from the cloud-based station using the publish–subscribe-based network protocol, as shown in Figure 8. For experiments on this multirobot system, data for 100 random images were simultaneously sent from each robot node to the cloud-based station and the resultant data were returned. The resultant data consisted of how many objects were in each image, how many were unknown and known, and the object class of the known objects. Figure 16 shows a schematic diagram for a single image in this experiment, and Table 5 shows the results.

**Figure 17:**
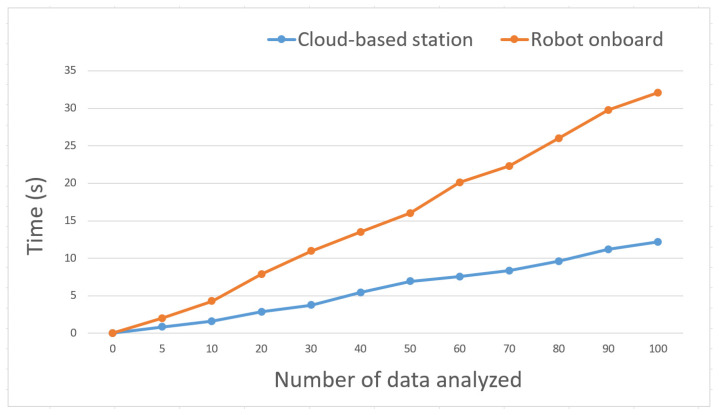
Computational power analysis between individual robot node and cloud-based station.

Providing high computational power and resources to each individual robot node is a difficult and expensive process. The proposed model reduces the need for robots’ onboard high computational power and resources by using the cloud-based station, which performs all data analysis and storage rather than performing on each individual robot onboard. To evaluate the computational power efficiency, we conducted an experiment where the entire system was implemented on an individual robot node (here on a raspberry Pi-4B) and compared it with the cloud-based implementation. In this experiment, a set of 100 images was used for analysis, and Figure 17 shows the results. The cloud-based method finished the data analysis for 100 images within 12.2 s; in contrast, robot onboard analysis finished in 32.1 s. This result shows that the proposed method reduced the burden of high computational power. Also, if we want to place the same computational power and resources onboard each robot, this increases the difficulty and expense.

Badhe et al. [34] adopted MobileNetV2 and some other network architectures for detecting objects from multiobject data. The SSDlite_mobilenetV2_coco model was trained using the COCO dataset. They obtained a test accuracy of 78% for the COCO-trained model.

Ma et al. [35] described the experimental outcomes and network architecture of ShuffleNet V2, which is also a lightweight CNN network architecture. They showed the model performance based on mean average precision and floating-point operations per second for the COCO dataset.

Mehta et al. [36] presented a lightweight network model named MobileViT for mobile vision tasks by combining CNNs and self-attention-based vision transformers. The author conducted an experiment using the COCO dataset and compared the performances with other CNN models. The method achieved better performances. 

To validate the performance of the proposed model, the object recognition performance was compared with those three state-of-the-art methods. Those models along with the proposed model were implemented with the “out” subset of COCO. Table 6 presents the performance comparison. In [34], the author used MobileNetV2 as a classification model. However, we used MobileNetV3 as an improved version of MobileNets. In [35], the author used ShuffleNetV2, which has a less accurate performance than that of MobileNetV3 [28]. In [36], the author used MobileViT but they did not consider unknown objects’ separation. The state-of-the-art models did not consider the handling of objects with unknown classes, which degraded their classification performance. Alternatively, the proposed model with unknown object detection used a one-class SVM and retrained the unknown objects using incremental learning to improve the object classification performance. The proposed model achieved 97.67% classification accuracy, mentioned as bold in Table 6.

The proposed model of this paper is very much effective and applicable to several real-world applications. However, besides the many advantages, it has some limitations.Our proposed model can efficiently use robot data with a cloud-based architecture, and also methods such as efficient unsupervised segmentation and incremental learning by separating unknown data, and is a lightweight classification method. We physically implemented the cloud-based architecture, unsupervised segmentation, and incremental-learning method and also presented the implementation process for easily implementing this architecture and our methods. In addition to mobile robot data analysis, management, and efficient multirobot communication, our proposed method can be reliably applied in other fields, such as the medical sector, product defect detection, surveillance, and autonomous driving. For example, in the medical sector, the proposed method can be applied to separate and segment patients’ unknown disease data and efficiently manage them with a cloud-based architecture to benefit many patients and doctors. Also, because all data are stored and managed by a cloud-based station, data security is reliably ensured by secure databases. Moreover, in other applications, image data processing, segmentation, and recognition are involved. So, the proposed model may reliably be applicable in robotics and other sensor data applications. Our segmentation algorithm is much more robust in terms of multiobject segmentation. However, it might be improved for more complex images or images with dark backgrounds. The incremental learning method still needs user intervention for the training process, which can be automated. These limitations of the proposed method will be investigated in our future work.

## 5. Conclusions

This paper proposed an efficient object segmentation model for separating multiple objects of robotic view-frame and SVDD-based unknown object detection for avoiding collisions between objects and obstacles in an environment for a mobile cloud–robot application. The SVDD-based one-class SVM outlier detection model helped to detect unknown objects to enhance the performance of object classification and detect unknown objects via retraining with an incremental learning method. A lightweight CNN model (MobileNetV3) was adopted to classify environmental objects using the incrementally trained model containing multiobject classes. An efficient cloud–robot architecture facilitated with cloud CNN, SVDD, and image segmentation algorithm supported high computational power, data storage, robot data sharing, and communication. In this research, cloud–robot architecture was implemented using Node-RED and a cloud environment and tested with a benchmark image dataset in a lab environment for validating the proposed model. This approach incrementally trains the one-class SVM and CNN models for new object-class adaptation and the implementation results show good outcomes. The most effective aspect of this unknown object detection is that the robot does not receive incorrect information. The performance of the known object classification using the CNN model is 97.67%, which leads to a better result. The performance of the proposed model was superior when compared with three state-of-the-art models. The proposed system can be useful for next-generation autonomous mobile robots.

## Figures and Tables

**Table 2:** Results and performance for each test case during implementation of one-class SVM and incremental learning.

Test Cases	Learning Condition	Known and Unknown Classes											
Case-1	Classes	person	chair	table	dog	stop sign	Unknown						
	Train (sample)	100	100	100	100	100							
	Test (sample)	50	50	50	50	50	200						
	Classified	Known class classified (244/250) 97.60%					195/200 98.5%						
Case-2	Classes	person	chair	table	dog	stop sign	cat	bottle	Unknown				
	Train (sample)	Already trained					100	100					
	Test (sample)	50	50	50	50	50	50	50	200				
	Classified	Known class classified (345/350) 98.57%							193/200 96.50%				
Case-3	Classes	person	chair	table	dog	stop sign	cat	bottle	potted plant	couch	Unknown		
	Train (sample)	Already trained							100	100			
	Test (sample)	50	50	50	50	50	50	50	50	50	300		
	Classified	Known class classified (441/450) 97.78%									294/300 98.00%		
Case-4	Classes	person	chair	table	dog	stop sign	cat	bottle	potted plant	couch	tv	microwave	teddy bear
	Train (sample)	Already trained									100	100	100
	Test (sample)	50	50	50	50	50	50	50	50	50	50	50	50
	Classified	Known class classified (587/600) 97.83%											

**Table 3:** Sample detection outcome for test cases 1 and 2 (where 1 = known and 0 = unknown).

Test Case—1			Test Case—2		
Object Class		Detection Outcome	Object Class		Detection Outcome
person	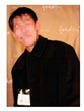	1	stop sign	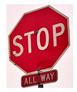	1
dog	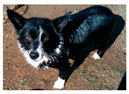	1	potted plant	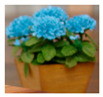	0
bottle	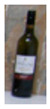	0	cat	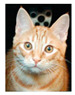	1
chair	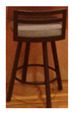	1	person	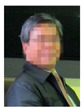	1

**Table 4:** Individual class performance (precision, recall, f1-score, support, and accuracy) for the trained one-class SVM models.

Class Name	Known/Unknown	Precision	Recall	f1-Score	Support	Class Accuracy
Person	0	1.00	0.98	0.99	50	99%
	1	0.99	1.00	1.00	100	
Chair	0	0.98	0.96	0.99	50	97.5%
	1	0.98	0.99	0.99	100	
Table	0	1.00	0.96	0.98	50	98%
	1	0.98	1.00	0.99	100	
Dog	0	0.94	0.98	0.96	50	97.5%
	1	0.99	0.97	0.98	100	
Stop sign	0	0.98	0.98	0.98	50	98.5%
	1	0.99	0.99	0.99	100	
Cat	0	1.00	1.00	1.00	50	100%
	1	1.00	1.00	1.00	100	
Bottle	0	1.00	1.00	1.00	50	100%
	1	1.00	1.00	1.00	100	
Potted plant	0	0.92	0.98	0.95	50	97%
	1	0.99	0.96	0.97	100	
Couch	0	0.94	0.96	0.95	50	96.5%
	1	0.98	0.97	0.97	100	
TV	0	1.00	0.98	0.99	50	99%
	1	0.99	1.00	1.00	100	
Microwave	0	1.00	0.94	0.97	50	97%
	1	0.97	1.00	0.99	100	
Teddy bear	0	0.96	0.96	0.96	50	96.99%
	1	0.98	0.98	0.98	100	

**Table 5:** Results for multiple robot nodes simultaneously sending data to the cloud-based station and receiving analysis information.

Robot Node	Sample Images (Sent)	Segmented Objects	Detected as Unknown	Detected as Known	Misclassified	Accuracy (%)	Execution Time(s)
R1	100	157	12	145	3	97.93	13.21
R2	100	149	11	138	1	99.27	12.85
R3	100	162	13	149	4	97.31	13.42
R4	100	155	9	146	3	97.94	12.69

**Table 6:** Comparison of proposed model and state-of-the-art algorithms based on classification accuracy.

Network Architecture	Accuracy (%)
SSDlite_mobilenetV2_coco [34]	89.61
ShuffleNet V2 [35]	91.42
MobileViT-XS [36]	94.08
**MobileNet V3 + one-class SVM [in proposed model]**	**97.67**

## Data Availability

Not applicable.
